# Nicotinamide Adenine Dinucleotide (NAD) Metabolism as a Relevant Target in Cancer

**DOI:** 10.3390/cells11172627

**Published:** 2022-08-24

**Authors:** Lola E. Navas, Amancio Carnero

**Affiliations:** 1 Instituto de Biomedicina de Sevilla, IBIS, Hospital Universitario Virgen del Rocío, Universidad de Sevilla, Consejo Superior de Investigaciones Científicas, 41013 Sevilla, Spain; 2 CIBERONC, Instituto de Salud Carlos III, 28029 Madrid, Spain

**Keywords:** nicotinamide adenine dinucleotide, NAD metabolism, therapeutic target, cancer

## Abstract

NAD+ is an important metabolite in cell homeostasis that acts as an essential cofactor in oxidation–reduction (redox) reactions in various energy production processes, such as the Krebs cycle, fatty acid oxidation, glycolysis and serine biosynthesis. Furthermore, high NAD+ levels are required since they also participate in many other nonredox molecular processes, such as DNA repair, posttranslational modifications, cell signalling, senescence, inflammatory responses and apoptosis. In these nonredox reactions, NAD+ is an ADP-ribose donor for enzymes such as sirtuins (SIRTs), poly-(ADP-ribose) polymerases (PARPs) and cyclic ADP-ribose (cADPRs). Therefore, to meet both redox and nonredox NAD+ demands, tumour cells must maintain high NAD+ levels, enhancing their synthesis mainly through the salvage pathway. NAMPT, the rate-limiting enzyme of this pathway, has been identified as an oncogene in some cancer types. Thus, NAMPT has been proposed as a suitable target for cancer therapy. NAMPT inhibition causes the depletion of NAD+ content in the cell, leading to the inhibition of ATP synthesis. This effect can cause a decrease in tumour cell proliferation and cell death, mainly by apoptosis. Therefore, in recent years, many specific inhibitors of NAMPT have been developed, and some of them are currently in clinical trials. Here we review the NAD metabolism as a cancer therapy target.

## 1. Nicotinamide Adenine Dinucleotide (NAD+)

**Figure 1:**
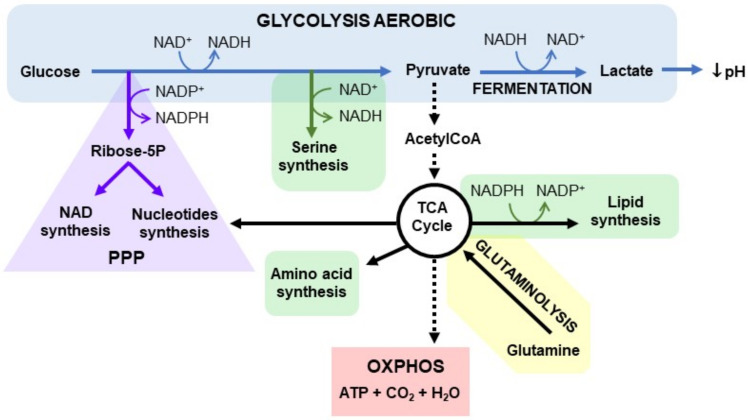
NAD+ metabolism. Tumour cells depend more on glycolysis (in blue) than on OXPHOS from the mitochondria (in dashed black). The pentose phosphate pathway (in purple), serine biosynthesis (in green), and fatty acid synthesis (in red) are highly activated in cancer and depend on glycolysis, which is the axis of cancer metabolism. Glutaminolysis (in brown) is also increased as the main source of nitrogen in the cell. The NAD+/NADH and NADP+/NADPH cofactors participate in all these pathways, allowing rapid energy production, the elimination of excess ROS, and the synthesis of macromolecules to support tumour proliferation and development.

NAD+ is a fundamental metabolite required for multiple oxidation–reduction (redox) reactions to maintain cellular homeostasis and energy production [1,2,3]. The NAD+ molecule is a dinucleotide composed of adenosine monophosphate (AMP) linked through a phosphate group to nicotinamide mononucleotide (NMN), which, in turn, is made up of the phosphate group, a ribose ring and nicotinamide [1,4]. The electrochemical properties of the nicotinamide moiety are essential for the classical redox functions of NAD+. Thus, the nicotinamide group can accept a hydride anion (H+, 2e−), reducing the molecule to NADH. In contrast, NADH is oxidised to NAD+ by donating that hydride anion [5,6]. Alternatively, the ribose ring of the AMP moiety of NAD+ can be phosphorylated by the enzyme NADK (NAD kinase), generating NADP+ (nicotinamide adenine dinucleotide phosphate), which can be reduced to NADPH [7]. All forms of NAD (NAD+, NADH, NADP+, NADPH) act as electron acceptors and donors in multiple redox reactions in the cell (Figure 1).

The NAD+ oxidised form is ubiquitous and the form most abundant in cells. However, its availability varies depending on intracellular factors, such as subcellular compartment, tissue and cell type, and intracellular glucose levels, but also on extracellular factors, such as caloric intake or expenditure, ageing or circadian rhythms. For example, it has been observed that free NAD+ concentrations in the nucleus and in the cytosol are similar since both compartments are connected, while the free NAD+ content in mitochondria is much higher (approximately 100 μM in nucleus/cytosol vs. 250–500 μM in mitochondria), ensuring a quick local regulation of NAD-dependent redox processes [8,9]. NAD+ concentration can also vary greatly during the day because of the string regulation by the circadian rhythm. The circadian clock complex CLOCK:BMAL1 induces the transcription of NAMPT, which is the limiting enzyme in the biosynthesis of NAD+, thus increasing the production of NAD+ every 24 h. SIRT1, which is an important NAD+-consuming factor, counteracts this effect by deacetylating BMAL1 and the promoter histones of other regulatory genes [10,11,12].

## 2. Redox Functions of NAD+

Initially, NAD+ was considered to have the role of a universal electron carrier in oxidation–reduction (redox) reactions of catabolic processes for energy generation in cells. One molecule of glucose during glycolysis will give rise to two NADH molecules, two ATP molecules, and two pyruvate molecules. Depending on the availability of oxygen, pyruvate can be reduced to lactate or oxidised to acetyl-CoA. In the absence of oxygen, the enzyme lactate dehydrogenase (LDH) reduces pyruvate to lactate using NADH as a cofactor, which is oxidised to regenerate NAD+. Conversely, in the presence of oxygen, pyruvate is oxidised to acetyl-CoA with the reduction of NAD+ to NADH [2,3,8]. Another process that also requires the reduction of NAD+ is the oxidation of fatty acids with the generation of acetyl-CoA [1,13]. In the mitochondria, after acetyl-CoA enters the Krebs cycle, more NAD+ is reduced to NADH by Krebs cycle enzymes. The mitochondrial NADH is oxidised by complex I (NADH:ubiquinone oxidoreductase) of the electron transport chain. Then, the electrons sequentially pass to ubiquinone, complex III, cytochrome C, and complex IV, generating a proton gradient across the inner mitochondrial membrane that ultimately leads to the production of more than 30 ATP molecules [1,2,3,8]. This process generates a large number of reactive oxygen species (ROS) that can inflict damage by oxidative reactions. Efficient mitochondrial metabolism requires an optimal NAD+/NADH ratio [14,15].

NADP+, NAD+ phosphorylated form and NADPH, reduced NADP+, are required for anabolic processes, including the synthesis of ribose-5-phosphate, used for nucleotide synthesis, via the PPP (pentose phosphate pathway) and lipid synthesis [16,17]. Furthermore, NADPH is essential to detoxify the high ROS levels produced in redox reactions [17]. The correct maintenance of redox homeostasis is an essential cellular process that requires a tight balance between the generation and elimination of ROS. This tight regulation is especially relevant in tumour cells, which have a higher production of ROS in general and need stricter control of this balance [18]. NADPH is also an important cofactor in the synthesis of fatty acids, which are necessary in the formation of the membrane during cell proliferation, using acetyl-CoA as a precursor [3] (Figure 1).

## 3. NAD+ as a Substrate in Nonredox Reactions

**Figure 2:**
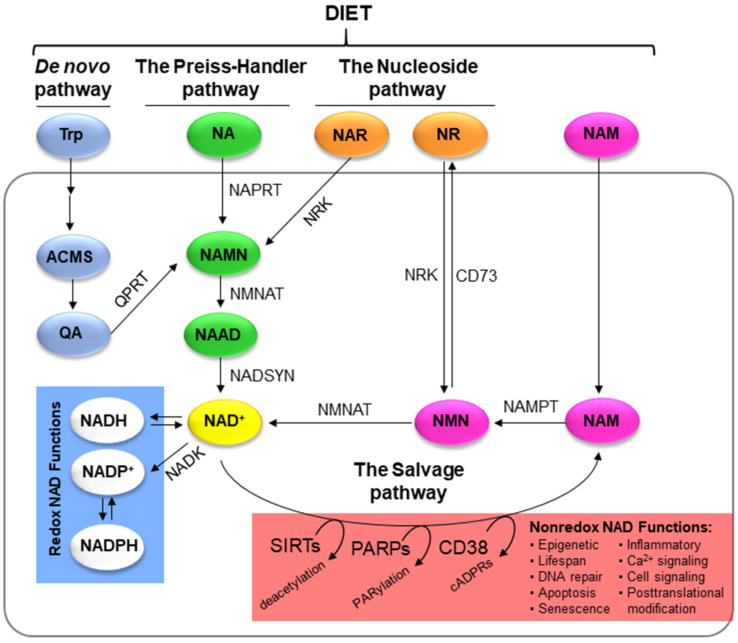
Different pathways in NAD+ metabolism. De novo pathway, the Preiss–Handler pathway, the salvage pathway and the nucleoside pathway are responsible for maintaining cellular NAD pools to be used in redox and non-redox reactions.

NAD+ and NADH can be reduced/oxidised in an almost unlimited manner [5]. However, the oxidised form, NAD+, is much more abundant than NADH, especially in the cytoplasm and nucleus (100-fold higher in those compartments, only 7 to 8-fold higher in the mitochondrial matrix) [19], highlighting the tight regulation of the process. High NAD+ levels are required since they participate in many other nonredox molecular processes, such as DNA repair, posttranslational modifications, cell signalling, senescence, inflammatory responses and apoptosis [1,2,4,6,17,20,21] In these nonredox reactions, NAD+ is a substrate for different types of enzyme families, mainly sirtuins (SIRTs), poly-(ADP-ribose) polymerases (PARPs) and cyclic ADP-ribose (cADPRSs). These enzymes use NAD+ as an ADP-ribose donor, releasing the catabolic product nicotinamide (NAM) [7,22] (Figure 2). The SIRT family consists of NAD+-dependent deacetylase enzymes that remove acetyl groups from lysine residues from proteins, such as histones, generating NAM and o-acetyl-ADP-ribose [22,23,24]. PARP family members transfer ADP-ribose long chain polymers from NAD+ to their target proteins involved in many different biological processes, such as metabolism, transcription or DNA repair [20]. The ectoenzymes CD38, CD157, CD39 and CD73 belong to the ADP-ribose synthases (cADPRS) family that consume NAD+ and NADP+, generating cyclic ADP-ribose (cADPR) or nicotinic acid adenine dinucleotide phosphate (NAADP), which act as secondary messengers of calcium signalling [19,23,25]. SIRTs, PARPs and cADPRSs are capable of coregulating each other, competing for the same subcellular NAD+ pool. SIRT1 can deacetylate PARP1 to inhibit its activity, thereby increasing the levels of NAD+, while PARP2 activity can inhibit the transcription of SIRTs [19,22,23]. Therefore, the activation of all these enzymes strongly depends on the availability of free NAD+. The importance of the role of NAD+ in these nonredox reactions has been well established, and recently, it has been suggested that NAD+ is the link connecting signal transduction and metabolism in physiological processes and many diseases [9].

## 4. Cancer and NAD+ Metabolism

Depending on internal genetic or epigenetic changes or microenvironmental conditions, cancer cells can reprogram their metabolism. This metabolic plasticity permits a high growth rate and survival with limited energy resources and adverse conditions. This metabolic plasticity rewiring allows an advantage during tumour progression and survival [26]. Higher ratios of the oxidised forms of NAD+ and NADP+ have been reported in tumour cells than in nontumor cells, suggesting an essential role for NAD+ in this plasticity [27]. Tumour cells are able to switch metabolism from OXPHOS to glycolysis, even under normal oxygen conditions (in a process known as the Warburg effect) [28,29]. Although OXPHOS metabolism generates energy in a more efficient way, producing 36 ATP molecules, tumour cells prefer glycolysis that generates less energy but in a faster way, producing two ATP molecules. It has been suggested that this fast energy production results in an important competitive advantage in sharing limited nutrients with the microenvironment and stromal cells [30,31]. However, this is not the only advantage of anaerobic glycolysis; it also allows tumour cells to synthesise macromolecules and counteract ROS production. In fact, part of the pyruvate that is formed is oxidised to acetyl-CoA to enter the Krebs cycle to produce the intermediate compounds of the cycle and use them as biosynthetic precursors. At the same time, these compounds are consumed and must be replenished primarily through the metabolism of glutamine, which is the main source of nitrogen in cells. Tumour cells depend on glutaminolysis for the synthesis of other molecules, such as nonessential amino acids, GSH, NAD+, NADPH and nucleic acids [29,31,32,33]. Some intermediates of glycolysis are also relevant for other processes promoted in cancer cells. For example, the PPP is essential for the synthesis of nucleic acids, DNA replication and the increase in tumour biomass [29,34]. In addition, the PPP is the main source of NADPH, which is required to reduce the ROS produced by accelerated metabolism, hypoxia or the accumulation of DNA damage in tumour cells. Radio- and chemotherapy also increase ROS levels, and in response, enzymes such as the PI3K/Akt/mTOR pathway, ATM, Ras or Src induce the expression of glucose-6-phosphate dehydrogenase (G6PDH), activating the PPP [16,34].

NAD+-dependent serine synthesis is another glycolysis-dependent pathway that is increased in cancer. The precursor of glycine and cysteine can be serine, which is involved in methionine and folic acid metabolism, redox balance, histone and DNA methylation and amino acid transport in the cells [35,36]. Metabolism in tumour cells depends highly on the PPP, serine biosynthesis and fatty acid synthesis. These metabolic pathways, which are dependent on NAD+ and NADP+, emerge from glycolysis, making NAD+ biosynthesis a main driver of cancer metabolism [1] (Figure 2).

## 5. NAD+ Biosynthesis

To meet the stringent NAD+ demands of redox and nonredox processes, NAD+ is generated from precursors such as tryptophan or vitamin B3 forms. These precursors are essential compounds that must be taken from the diet for the synthesis of NAD+. In fact, a chronic deficiency of these precursors can cause pellagra, an illness that can even lead to death. Interestingly, the essential role of NAD+ in cells was discovered when pellagra treatment was being investigated in the 1920s [3,19]. From these precursors, cells synthesise NAD+ by three pathways: the de novo pathway, the Preiss–Handler pathway and the nucleoside pathway. Additionally, cells can recycle the catabolic product released by NAD+-consuming enzymes (SIRTs, PARPs and cADPRs) and reconstitute the molecule through the salvage pathway, which is the most frequently used option for cells [8,9,10,11,37].

### 5.1. De Novo Pathway

NAD+ can be synthesised de novo from tryptophan, which is an essential amino acid that must be incorporated daily through the diet. In humans, this pathway, also called the kynurenine pathway, is present mainly in the liver and consists of several steps [9]. The limiting metabolite is quinolinic acid (QA). Its precursor, 2-amino-3-carboxymuconic acid semialdehyde (ACMS), can be oxidised to picolinic acid (PA) by the enzyme 2-amino-3-carboxymuconic acid semialdehyde decarboxylase (ACMSD) or enter a spontaneous cyclization, giving rise to QA. Compound QA is only formed when the ACMSD enzyme becomes saturated. Once formed, QA enters the Preiss–Handler pathway through the enzyme quinolinate phosphoribosyltransferase (QPRT) to continue NAD+ synthesis [3,7]. Trp is also required for the synthesis of other essential molecules, such as serotonin and melatonin, and approximately only 1.66% of dietary Trp is used for NAD+ biosynthesis. This low efficiency may explain why the de novo pathway is not the main source of NAD+ in humans.

### 5.2. The Preiss–Handler Pathway

NAD+ can also be synthesised from niacin and nicotinic acid (NA) present in the diet. Nicotinic acid phosphoribosyltransferase (NAPRT) is the rate-limiting enzyme in this pathway converting nicotinic acid to the intermediate nicotinic acid mononucleotide (NAMN). Consequently, NAMN is later transformed into NAAD by the enzyme NMNAT. This enzyme has three isoforms with different subcellular localizations: NMNAT-1 is found in the nucleus, NMNAT-2 is mainly present in the golgi apparatus and the cytosol and NMNAT3 is located in the cytosol and mitochondria [3]. Finally, the enzyme NAD synthetase (NADSYN) uses glutamine as a donor to amidate NAAD and form NAD+ [37].

### 5.3. The Salvage Pathway

Taking into account that the half-life of NAD+ is approximately 4–10 h and that the average daily intake of vitamin B3 is 15 mg, which does not cover physiological needs (for example, the liver needs approximately 8 g per day), cells have no choice but to recompose the NAD+ molecule through the salvage pathway [3]. Nicotinamide (NAM), the catabolic product released by NAD+-consuming enzymes, is recycled in two stages. The first step is the rate-limiting reaction of the pathway catalysed by NAMPT, transforming NAM to nicotinamide mononucleotide (NMN) in the presence of magnesium (Mg^2+^) and phosphoribosyl pyrophosphate (PRPP). In the second stage, the enzyme NMNAT reconstitutes NAD+ from NMN [38,39,40,41,42,43]. This pathway, the salvage pathway, is the main source of NAD+ generated in mammalian cells due to its high production efficiency, and less than 1% NAD+ is lost daily due to efficient recycling. This might explain why cells are completely dependent on this pathway [1,3,7,42,43].

### 5.4. The Nucleoside Pathway

Nicotinamide riboside (NR) and nicotinic acid riboside (NAR) are other NAD+ precursors that are acquired from the diet. Once they enter the body, the enzyme nicotinamide ribose kinase (NRK) is responsible for phosphorylating NAR, giving rise to NAMN (an intermediate in the Preiss–Handler pathway) and converting NR directly to NMN, bypassing the limiting reaction catalysed by NAMPT of the salvage route [7]. Until recently, it was thought that the cell membrane was impermeable to extracellular NMN generated by extracellular NAMPT and CD38. Thus, NMN must first be dephosphorylated to NR by the enzyme CD73, and then NR enters the cell through nucleoside transporters (ENTs) to equilibrate the pools [3]. However, Grozio et al. recently identified an NMN transporter in the cell membrane called Slc12a8 [44,45]. However, the expression of Slc12a8 is limited to the intestine and others have questioned its ability to transport NMN [46].

## 6. Nicotinamide Phosphoribosyltransferase (NAMPT)

Nicotinamide phosphoribosyltransferase (NAMPT), in addition to being the rate-limiting enzyme of the NAD+ salvage pathway, also acts as a cytokine-modulating inflammatory, metabolic, and immune responses [47,48]. In fact, NAMPT was discovered in lymphocytes in human peripheral blood and was initially called pre-B colony-stimulating factor (PBEF) since it is involved in the maturation of B lymphocytes in the presence of interleukin 7 (IL-7) and stem cell factor (SCF) [49,50]. This extracellular form of NAMPT is commonly called visfatin to differentiate it from the intracellular form. Visfatin is secreted mainly by adipocytes, macrophages, lymphocytes and inflamed endothelial cells and is involved in inflammatory processes and metabolic disorders such as obesity and cancer. For example, adipocytes release visfatin into the extracellular medium, where it exerts an insulin-mimetic function and regulates glucose and visceral fat levels related to obesity. In fact, high levels of visfatin have been detected in obese people [47,51]. Interestingly, the visfatin protein sequence lacks a secretion signal, so the protein is thought to be the same as intracellular NAMPT released after cell lysis [47,52]. The intracellular form of NAMPT is expressed in all tissues, mainly in those that require more energy, such as bone marrow, liver and muscle. Within the cell, NAMPT is located mainly in the cytoplasm and nucleus [43,50].

**Figure 3:**
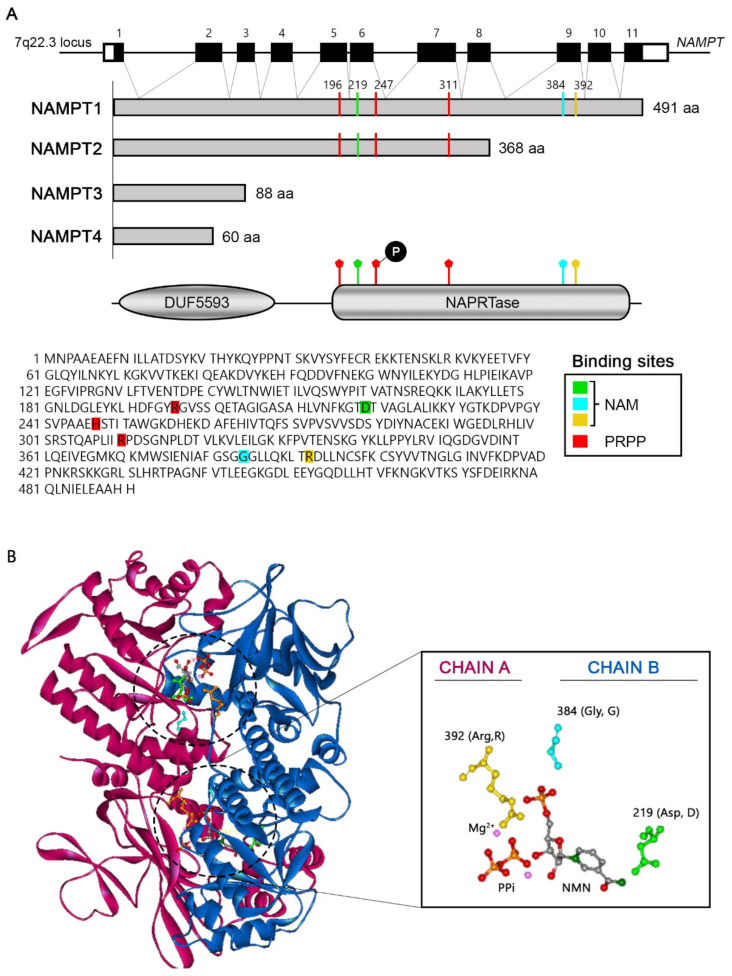
Structure of the NAMPT gene and protein. (**A**) The NAMPT gene contains 11 exons and 10 introns that are translated into 4 possible variants by alternative splicing or splicing. NAMPT1 is the predominant variant and the only one that has enzymatic activity. The protein contains 2 domains, DUF5593 and NAPRTase, in which the amino acids that form the catalytic site of NAM (D219, G384 and R392) and PRPP (R196, H247 and R311) are located. H247 aa must first be phosphorylated by ATP hydrolysis for NMN synthesis to take place. (**B**) Crystal structure of the NAMPT functional homodimer (3DHF; https://www.rcsb.org/structure/3DHF, Accessed on 1 April 2022). Catalytic sites are marked with dashed circles.

The NAMPT gene is located on chromosome 7 at the 7q22.3 locus and contains 11 exons and 10 introns [43,49,53]. Approximately 19 messenger RNAs and 14 variants have been predicted to be produced through alternative splicing (Figure 3). Of all these variants, only four likely produce proteins: variant 1 with a size of 491 amino acids (aa), variant 2 with 368 aa, variant 3 with 88 aa and variant 4 with 60 aa. The predominant variant is variant 1, NAMPT1, which is the only variant that possesses the enzymatic activity necessary for the conversion of NAM to NMN [53,54]. The protein contains two recognised domains: DUF5593, which comprises the N-terminal aa 10–116 and whose function is unknown, and NAPRTase, which comprises aa 188–466 and is responsible for the enzymatic activity of the protein [55,56]. The amino acids that make up the catalytic site are found at positions D219 (aspartic acid), G384 (glycine) and R392 (arginine). Additionally, amino acids R196 (arginine), H247 (histidine) and R311 (arginine) form the binding site of cofactor PRPP [57] (Figure 3A). To be functional, NAMPT1 must form a homodimer with two catalytic sites, each of which is made up of two aa from one chain and one aa from the other chain [58,59] (Figure 3B) In mammals, it appears that NAMPT can be activated by autophosphorylation. Thus, for NMN synthesis to occur, histidine 247 must first be phosphorylated by ATP hydrolysis and in the presence of Mg^2+^. Phosphorylated NAMPT has been shown to be more active than its unphosphorylated form. Once the enzyme is active, its substrates bind, first PRPP and then NAM, each to its corresponding binding site [53,60,61].

The other variants that have been predicted (NAMPT2, NAMPT3 and NAMPT4) do not contain these amino acids required for enzymatic activity assigned to NAMPT1, and it is currently unknown whether they have any function in the cell. In the present work, NAMPT will be mentioned, referring exclusively to the variant NAMPT1.

## 7. NAMPT in Cancer

To meet NAD+ demands, tumour cells must maintain high NAD+ levels, enhancing their synthesis mainly through the salvage pathway. The rate-limiting enzyme of this pathway, NAMPT, has been identified as an oncogene in some cancer types. In fact, increased NAMPT levels have been found in both haematologic malignancies [62,63] and various solid tumours, including colon [40], prostate [64], breast [65], thyroid [66], gastric [67] and glioblastoma tumours [41,42]. NAMPT is capable of increasing the tumorigenic properties of cells, such as cell proliferation, clone formation and resistance to apoptosis, thus allowing tumour initiation and progression [40,41]. The expression of NAMPT also correlates with the clinical data of cancer patients in some tumours. For example, in stomach, colon and glioblastoma cancer, high levels of NAMPT correlate with aggressive tumour stages, metastasis, treatment resistance and worse prognosis [40,41,64,67]. It also activates the secretion of interleukin 6 (IL-6), which induces the STAT3 signalling pathway, which favours the process of new blood vessel formation [62,68,69].

Visfatin (or extracellular NAMPT) is also implicated in cancer and its role is currently being studied. Although the exact molecular mechanism of visfatin remains unknown, its role is currently being studied. In fact, high levels of serum visfatin have been detected in patients with variant types of cancer, including postmenopausal breast cancer, endometrial cancer, gastric cancer and hepatocellular carcinoma. Visfatin has been proposed as a diagnostic marker in cancer and it is associated with poor patient survival [46,70,71,72]. Visfatin acts as a tumour cytokine promoting proliferation, neovascularization and metastasis in cancer. It has been described to regulate the function of adhesion molecules such as ICAM-1 and VCAM-1 through the activation of NF-κB in human endothelial cells [46]. It also upregulates matrix metalloproteinase, such as MMP-2 and MMP-9, and vascular endothelial growth factors, promoting angiogenesis via the STAT3/IL-6 signaling pathway [68,73]. Recently, it has been described that Visfatin can be secreted from cells via exosomes, which can promote the inflammatory and tumor microenvironment [74,75]. Similar to other adipocytokines (e.g., leptin, adiponectin and resistin), visfatin contributes to autocrine, paracrine and endocrine interactions between tumour cells and the microenvironment [46,76]. In breast cancer, it has been reported that visfatin induces THP-1 differentiation into M2 macrophages suppressing the immune response promoting tumor progression. Moreover, M2 macrophages secrete CXCL1 which elevated visfatin via a positive feedback loop. Visfatin/M2/CXCL1 contribute to tumor migration and invasion [77].

On the other hand, the exact molecular mechanism of visfatin remains unclear and its cellular receptor has been not found. However, visfatin presents insulin-mimetic effects, and it has been described that visfatin binds to and activates the insulin receptor of hepatocytes, myocytes and adipocytes [52]. There is controversy regarding if visfatin has the enzymatic activity as its intracellular form. Although the dimeric conformation of visfatin has been elucidated, it has been difficult to detect all of its substrates (PRPP, NAM and ATP) in serum [47,75]. Moreover, the effects of visfatin do not depend on the presence of NAM, nor are they reproducible by NMN addition or blocked by NAMPT inhibitors [67,78]. Lastly, the effects of visfatin are independent of the intracellular enzymatic activity of NAMPT.

## 8. Other NAD+-Dependent Enzymes in Cancer

NAMPT overexpression generates an increase in NAD+ content in tumour cells, which enhances the activity of NAD+-dependent and NAD+-consuming enzymes (SIRTs, PARPs and cADPRSs). The oncogenic role of NAMPT can be explained through the activation of these enzymes.

### 8.1. SIRTs in Cancer

The sirtuin family (SIRT1–7) regulates the activity of histones, DNA repair and transcription and other signalling proteins by deacetylating several substrates. Therefore, its role as a tumour suppressor or oncogene has been described to be dependent on the type of cancer [22,23,79]. The best characterised member is SIRT1, which is associated with longevity. Resveratrol, a powerful antioxidant and antiaging compound, is capable of increasing NAD+ levels, inducing the activation of SIRT1 [22]. This enzyme is also activated by caloric restriction, which reduces the risk of cancer [2,16]. SIRT1 activity is highly dependent on the NAD+/NADH ratio, rather than only the levels of NAD+, and can be inhibited by high levels of NAM [28,80] or by DBC1, an endogenous SIRT1 inhibitor. Many studies suggest that SIRT1 also acts as a tumour suppressor. SIRT1 has been shown to inhibit the alpha subunit of hypoxia-inducible factor 1 (HIF-1α), one of the factors that can activate glycolysis [28]. However, high levels of SIRT1 were found in some tumours, in which NAMPT activates SIRT1 expression by increasing NAD+ levels and decreasing NAM [69,81]. SIRT1 regulates other transcription factors, such as p53, FOXO3, E2F1, c-MYC and NF-κB. P53 regulates cell cycle arrest and apoptosis and can also inhibit G6PDH in the pentose phosphate pathway. SIRT1 can deacetylate and inhibit p53, thus allowing survival by ensuring the production of NADPH and ribose-5-P [17,82]. The proto-oncogene C-MYC can induce NAMPT expression and inhibit DBC1, thereby activating SIRT1. In turn, SIRT1 activates c-MYC by deacetylation, generating a positive feedback loop [29,81]. In addition, SIRT1 activates FOXO3 by deacetylation, contributing to resistance to oxidative stress [64,83]. Thus, the oncogenic role of SIRT1 depends on NAD+ levels. The other members of the sirtuin family, SIRT2–7, similar to SIRT1, can also act as oncogenes or tumour suppressors according to the type of tumour.

### 8.2. PARPs in Cancer

PARP1, known as the guardian of DNA integrity, is the most studied member of the PARP family [84]. The PARP1 enzyme is activated in response to DNA damage, requiring high levels of NAD+ in the cell, and is thus dependent on NAMPT activity. Excessive activation of PARP1, due to the action of genotoxic agents, for example, dramatically reduces ATP and NAD+ pools, regulating sirtuin activity and, therefore, cellular homeostasis. If the DNA damage is not repairable, PARP1 promotes apoptosis through the activation of p53 under normal conditions. However, PARP1 can also regulate oncogenic transcriptional factors. Depending on the context, PARP1 has been reported to act as an oncogene or tumour suppressor [17,84]. Most tumours overexpress PARP1 in response to increased DNA damage aggravated by radiation and chemotherapy treatment. In fact, high levels of PARP1 have been found in breast, ovarian and lung cancers and non-Hodgkin lymphoma [43,84]. In colon cancer, PARP1 has been found to have a dual oncogenic and suppressive function [81,85]. In addition to DNA damage, PARP1 is involved in other physiological cell processes, such as proliferation, cell cycle regulation, gene transcription, inflammation and cell fate. PARP1 regulates ERK, allowing the activation of proangiogenic and metastatic factors. PARP1 also regulates the transcription factor E2F1, which reduces apoptosis in tumour cells [86,87]. Inhibition of PARP1 by olaparib has been tested in clinical trials for tumours of different origins. Pharmacologically, tumour cells are in general more sensitive to PARP1 inhibition than healthy cells. This sensitivity may be because tumour cells lack alternative DNA repair mechanisms. However, resistance to PARP1 inhibitors has been observed [16].

### 8.3. cADPRSs in Cancer

CD38, belonging to the cADPRSs family of ectoenzymes, hydrolyzes NAD+ to produce cADPRs, which is involved in Ca^2+^ mobilization, regulation of the cell cycle and insulin signalling [17,88]. CD38 is the largest consumer of NAD+ in mammalian cells, since it requires 100 molecules of NAD+ to generate only one molecule of cADPR. For that reason, CD38 has been proposed as an important regulator of intracellular NAD+ levels. As we age, the levels of NAD+ decline sharply. It has been shown that this depletion is partly due to the increased expression of CD38 that occurs with age [85,89]. CD38 is a transmembrane protein whose C-terminal catalytic domain has two opposite orientations and, therefore, is capable of consuming both intracellular and extracellular NAD+ [86,90]. CD38 also generates NAM by metabolizing extracellular NMN, which is converted to NAD+ via visfatin or NAMPT and NMNAT after crossing the cell membrane [1,85,89]. CD38 has been described as a marker of negative prognosis in CLL and as a diagnostic cell marker in multiple myeloma [16,91]. It has also been observed that CD38 expression levels can modify the sensitivity and resistance of pancreatic cancer cells to the NAMPT inhibitor FK866. However, the functional relationships between NAMPT, NAD+ and CD38 are not yet well known [83,92]. CD73, another member of the cADPRS family, catalyses the conversion of extracellular NMN to NR, which is necessary to aid in the biosynthesis of intracellular NAD+ [89,93]. In addition, CD73 has a relevant role in the migration and invasion of tumour cells, acting as an adhesion molecule [90,94].

### 8.4. NAPRT in Cancer

High levels of NAPRT and NAMPT have been found in ovarian, prostate and pancreatic cancers [63,95,96], while in glioblastoma, neuroblastoma, chondrosarcoma, leukaemia and gastric and colon cancers, low levels of NAMPT and low levels of NAPRT have been found [63,97,98,99,100]. Comparing the pattern of expression of the NAPRT and NAMPT genes in human tumour cells with nontumor cells, there is high variability in the levels of NAPRT mRNA and protein in tumour cells [97,101]. This high expression variability depends on the tissue of origin. Thus, tumours from tissues with high NAPRT expression will have higher NAPRT amplification, and conversely, tumours from tissues with low NAPRT expression will be dependent on NAMPT activity [96,100,102].

NAPRT-negative tumours usually present with mutations in isocitrate dehydrogenase 1 and 2 (IDH1/2) and protein phosphatase 1D (PPM1D). IDH1/2 mutants use NADPH to convert α-ketoglutarate to D-2-hydroxyglutarate (D-2HG), which acts as an oncometabolite inhibiting NAPRT expression by the hypermethylation of its promoter [98,103,104]. PPM1D is an oncogene that is involved in the DNA damage response and checkpoints of the cell cycle. PPM1D mutants also lead to the inhibition of the NAPRT promoter by the methylation of CpG islands [101,105]. The inhibition of the Preiss–Handler pathway by NAPRT forces tumour cells to rely entirely on the salvage route. Therefore, NAMPT is usually overexpressed in these tumours since their cells rely on its activity to meet the demands for NAD+ [100,104]. In fact, cells with IDH1/2 and PPM1D mutations are more sensitive to treatment with NAMPT inhibitors due to the total depletion of NAD+ [103,104,105]. In tumours with high levels of NAPRT, tumour cells may have a better supply of NAD+, which allows for the activation of NAD+-dependent enzymes, including PARP1, which are required to protect against DNA damage and oxidative stress [106,107]. Overexpression of NAPRT may contribute to resistance to treatment with NAMPT inhibitors or alkylating agents that cause DNA damage. In vivo, xenografts have been shown to be less sensitive to the NAMPT inhibitor FK866 if NAPRT is overexpressed, whereas they are more sensitive when NAPRT is inhibited [100,106]. NAPRT can be inhibited by compounds such as 2-hydroxynicotinic acid (2-HNA). 2-HNA is an analogue of nicotinic acid that competitively blocks the enzyme [108,109]. It has been observed that this compound is capable of potentiating the cytotoxic effect of the NAMPT inhibitor FK866. Therefore, the ability to predict the response to NAMPT inhibitor treatments by low levels of NAPRT depends on the type of cancer [100,106].

## 9. NAMPT in Cancer Stem Cells (CSCs)

**Figure 4:**
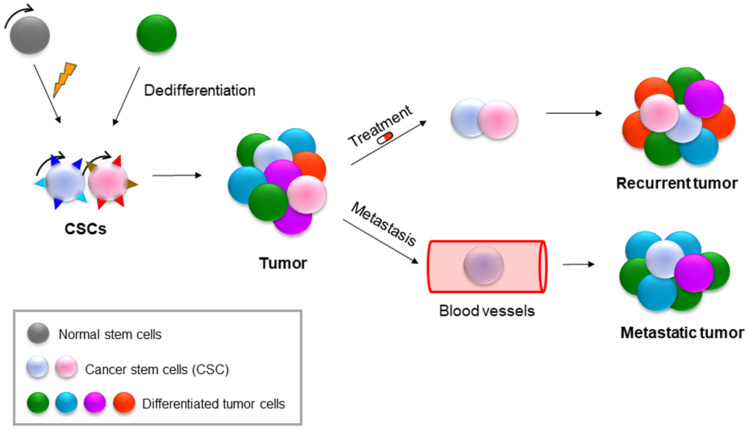
Brief scheme of CSC contribution to recurrence and metastasis.

Tumours are highly heterogeneous entities that are organised hierarchically into different subgroups of tumour cells. Cancer stem cells (CSCs) are at the top of this hierarchy. CSCs are a small subpopulation of cells with the ability to self-renew and differentiate into the different types of cells that form the bulk of the tumour. Similar to normal stem cells, these CSCs can self-renew and differentiate, but they do so in an uncontrolled manner. CSCs are responsible for tumour initiation and progression and for metastasis and resistance to therapies. CSCs can originate from normal stem cells or from differentiated tumour cells that are capable of acquiring stem cell capabilities through dedifferentiation mechanisms [110,111,112,113]. (Figure 4) The dedifferentiation process is similar to the process of reprogramming somatic cells into pluripotent cells (iPSCs), which is induced through the activation of some pluripotency transcription factors, such as OCT3/4, SOX2, KLF4 and c-MYC [114,115]. The interconversion capacity between both phenotypes, different cells and stem/pluripotent cells is known as cell plasticity. The cellular microenvironment and some signalling pathways activate and regulate this cellular plasticity. Some of the main pathways that regulate CSCs are Notch, Hippo, SHH, PI3K, WNT and NF-κB [110,113,116].

Both CsCs and iPSCs can show an active Warburg metabolic effect, which induces the hypoxic state necessary for the maintenance of the stem cell phenotype. Thus, the stem cell phenotype or pluripotency can be highly glycolytic when the cells are actively dividing [110,117,118]. However, much controversy exists regarding the typical metabolic profile of CSCs. In general, CSCs are in the G0 phase, or quiescence, a state in which they consume oxygen and depend mainly on OXPHOS metabolism, by which they obtain a greater antioxidant response to compensate for the high levels of ROS. This slow metabolism allows them to survive conventional therapies that mainly attack cells with high rates of proliferation. As sole survivors, CSCs change from their quiescent state to a more proliferative and glycolytic state, thus regenerating the tumour again [111,117,119]. In recent years, research has focused on searching for surface markers and transcriptional signatures specific to the CSC phenotype.

CSCs express specific antigens on their surface, similar to normal stem cells. These surface markers that characterise CSCs depend largely on the type of cancer, and the best known are CD44+CD24− and ALDH in breast cancer, CD44+ and CD133+ in colon and gastric cancer, CD34+CD38− in leukaemia, CD133+ in glioblastoma and sarcoma and CD13/CD45/CD90 in liver cancer [120,121]. However, the CSC population presents a high degree of heterogeneity, with different subgroups or categories even in the same tumour [110,113,116]. Finding new markers and genes that help us to identify CSC populations is vital to developing new and more specific therapeutic strategies. NAMPT enriches the CSC population of the tumour mainly through the salvage route, thus allowing an adequate supply of NAD+ [42,43]. NAMPT and NAD+ are involved in the processes of pluripotency and dedifferentiation to CSCs. It has been observed that media supplementation with NAM can prevent senescence and apoptosis and promote reprogramming and iPSC generation. Furthermore, reduced NAD+ levels in human stem cells can cause spontaneous differentiation and apoptosis [116,122]. The autofluorescence emitted by the NADH reduced form can be quantified by cytometry, and a subpopulation with high NADH has been related to the CD133+ subpopulation of CSCs in glioblastoma [123,124]. NAMPT not only increases tumorigenic properties but also allows the acquisition of the properties and phenotype of CSCs through the activation of OSKM factors, including SOX2, OCT4, KLF4 and NANOG [40,41]. In addition, NAMPT can regulate the canonical WNT pathway since the inhibition of NAMPT increases the levels of Axin, degrading β-catenin, which decreases tumorigenesis. The addition of NMN, the product of NAMPT, reverses the degradation of β-catenin and reduces the adhesion of mesenchymal cells, allowing them transform into cells with migratory and invasive capacities. NAMPT activates several transcription factors involved in EMT, such as SNAI1, TWIST and FOXC2, favouring tumour invasion and metastasis [40,41].

We found high levels of NAMPT expression in the tumorspheres generated from our head and neck squamous cell carcinoma (HNSCC) cell lines. NAMPT overexpression favoured the formation of clones and increased the expression levels of some genes involved in the regulation of stem cell pluripotency, such as SOX2 and NANOG, in HNSCC. However, in general, the reduction in NAMPT reduced the capacity for proliferation, clone formation and growth in conditions of competition with other cells and the in vivo growth of tumours generated in xenotransplantation models [1,2,40,41,42,43]. All these data indicate that NAMPT could be involved in the process of tumorigenesis in HNSCC.

NAMPT is a possible tumour and CSC marker in HNSCC. In several types of cancer, it has been described that NAMPT is capable of enriching the CSC population of the tumour through the processes of pluripotency and dedifferentiation, a mechanism by which cells acquire the properties and increase phenotype of tumour stem cells [40,41,43].

In addition, we found several genes that positively correlated with NAMPT overexpression from cells in the TCGA public database of HNSCC patients. These genes were *CDH1*, *DESI1*, *MAP1B*, *TMEFF1*, *OCLN*, *TCF4*, *SMAD2*, *TFRC*, *AHNAK*, *PATP4A1*, *MSN*, *STEAP1*, *PGK1* and *F11R* [1,2].

CSCs are thought to be responsible for resistance to radiotherapy and chemotherapy and the recurrence rate of tumours in patients [110,111,112]. In the TCGA database, we found that high levels of NAMPT correlated with worse survival in patients with HNSCC. Therefore, NAMPT could also be an indicator of poor prognosis in HNSCC, as occurs in stomach cancer, colon cancer and glioblastoma [40,41,64,67].

## 10. NAMPT as a Therapeutic Strategy

**Figure 5:**
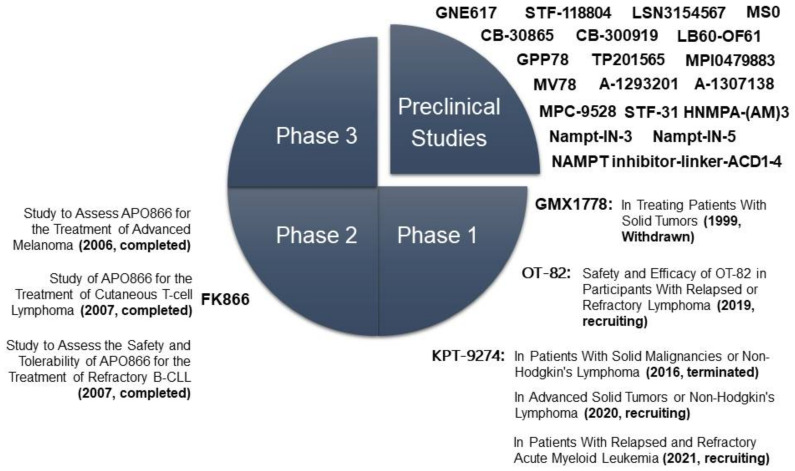
Scheme of the proliferation of NAMPT inhibitors in preclinical or clinical stages.

NAMPT inhibition causes the depletion of NAD+ content in the cell, leading to the inhibition of ATP synthesis. This effect can cause a decrease in tumour cell proliferation and cell death, mainly by apoptosis. Therefore, in recent years, many specific inhibitors of NAMPT have been developed, and some of them are currently in clinical trials [1,2,3,4,5,6,7,8,9,10,11,12,13,14,15,16,17,18,19,20,21,22,23,24,25,26,27,28,29,30,31,32,33,34,35,36,37,38,39,40,41,42,43,62,83,125,126] (Figure 5, Table 1 and Table 2) The first inhibitor to be developed, being the most studied both in vitro and in vivo, was FK866/APO866, a partially noncompetitive inhibitor that binds to the interface between the two subunits of the NAMPT homodimer, partially covering the binding site of NAM [57]. NAMPT inhibition by FK866 can increase ROS levels and decrease glucose consumption, ATP production, fatty acid synthesis and, of course, NAD+ levels, negatively affecting the activity of all NAD+-dependent enzymes, such as SIRTs and PARPs [79]. FK866 treatment does not seem to affect mitochondrial NAD+ content; therefore, tumour cells could be more sensitive than nontumor cells [59]. In addition, FK866 cytotoxicity appears to be greater in some tumour types that express higher levels of NAMPT and are relatively more dependent on glycolysis [83,102]. As of 2007, FK866 is in phase 2 clinical trials (clinicaltrials.gov; NCT00435084, NCT00431912, NCT00432107). To palliate the adverse effects of FK866, it has been tried at suboptimal doses in combination with other drugs, such as sirtinol (SIRT1 inhibitor) and olaparib (PARP1 inhibitor) [40,41,97] Another very specific NAMPT inhibitor studied is GMX1778/CHS-828, which is administered orally. The GMX1777 prodrug is a cyanoguanidine compound that is rapidly converted to its active form GMX1778, which acts as both an inhibitor and a substrate. GMX1778 phosphorylated by NAMPT accumulates inside tumour cells, enhancing its antitumor effect [93,97]. GMX1778 was in phase 1 clinical trials for solid tumours (clinicaltrials.gov, Accessed on 1 April 2022; NCT00003979); however, it was withdrawn due to its toxic effects, mainly thrombocytopenia and gastrointestinal symptoms [127]. CB30865 and its analogue CB300919 were initially developed to inhibit thymidylate synthase (TS), but it was later found that this protein was not its target, although the compounds had a potent cytotoxic effect [128]. Years later, it was discovered that the target protein was NAMPT [129]; however, due to solubility problems, these inhibitors were not used in clinical trials [130]. The next generation of inhibitors showed better pharmacological properties, including oral administration, and higher specificity against their NAMPT target. Among these inhibitors are GNE617 and its analogues GNE618, GNE643 and GNE875, which are small molecules for oral administration that demonstrated high efficacy in vivo and were able to reduce the growth of NAPRT-deficient xenotransplants. However, they also showed some cardiac, retinal and gastrointestinal toxicity [131,132,133]. Other inhibitors are MPC-9528 and its analogue MPI0479883, which showed a potent inhibition of tumour progression in fibrosarcoma xenografts [134]. STF-118804 also showed efficacy in several cell lines and in acute lymphoblastic leukaemia xenografts [135]. TP201565, a potent analogue of GMX1778, showed cytotoxic effects in several cell lines in vitro [136].

However, despite the powerful antitumor effect shown by all these NAMPT inhibitors, they have not entered clinical trials because of toxicity and solubility problems due to their lipophilic chemical structures. For this reason, the strategy of incorporating polar and ionizable groups into their structures has been adopted to improve solubility. This approach, in addition to reducing toxicity, would reduce the permeability of these compounds through the cell membrane so that they could also act on the extracellular form of NAMPT (eNAMPT) [154]. GPP78 and MV78 are two analogues of FK866 that have served mainly as templates for the development of new, more soluble inhibitors [139,141,155]. MS0 is also an analogue of FK866 that was found to have less effective in vitro activity [145], but it served as a template for the development of better inhibitors (compounds MS1–31) [146]. LB-60-OF61, one of the latest inhibitors developed, has shown a high efficacy in cell lines that overexpress the MYC oncogene [138], which regulates and activates the expression of NAMPT and inhibits DBC1 [81], the endogenous inhibitor of SIRT1. Trans-3-(pyridin-3-yl)acrylamide-sulfonamide compounds are potent inhibitors that have shown an antiproliferative effect at subnanomolar doses in various cell lines in vitro [142]. A-1293201 and A-1307138 are two inhibitors that lack the aromatic nitrogen group mimetic to that of the natural substrate NAM. In the study, it was observed that these inhibitors maintained their antitumor effect despite not being phosphorylated by the target enzyme NAMPT, demonstrating that the formation of the phosphorylated adduct was not essential for the functionality of NAMPT inhibitors [143]. Depletion of NAD+ can be very toxic to nontumor cells, so using NAD+ precursors (e.g., nicotinic acid) as adjuvants has been considered [63,96]. Several studies have found that the coadministration of nicotinic acid (NA) with some NAMPT inhibitors (GMX1778, GNE617, LSN3154567, MPC-9528 and STF-31) helps prevent toxic effects, such as haematologic and retinal toxicity in rodents, while maintaining drug efficacy. However, the inhibitory effect was suppressed in NAPRT-positive tumours, that is, tumours that overexpressed this enzyme. Therefore, the coadministration of NA and NAMPT inhibitors has been proposed as a new therapeutic strategy, but only in tumours deficient in NAPRT [97,134,137,147]. Moreover, it has been observed that treatment with NAMPT inhibitors combined with other drugs, such as PARP inhibitors, may be useful to improve the efficacy of the inhibitory effect and reduce toxicity [153,154]. Therefore, the development of inhibitors with dual therapeutic targets could be a good therapeutic strategy. An example would be the compound STF-31, which is a double inhibitor of NAMPT and of the glucose transporter GLUT1. This additional inhibitory effect could be beneficial in triggering cell death in tumours with a high rate of glucose consumption. In fact, it has been observed that STF-31 is capable of selectively attacking renal carcinoma cells deficient in the von Hippel–Lindau (VHL) tumour suppressor gene [147,156]. Nampt-IN-3 is a double inhibitor of NAMPT and histone deacetylase (HDAC) enzymes, an enzyme family to which sirtuins belong [148]. This compound could be useful in tumours in which sirtuins act as oncogenes. Nampt-IN-5 is a NAMPT inhibitor that also acts on CYP3A4 activity, enhancing its cytotoxic effect both in vitro and in xenograft models [149]. In addition, new NAMPT inhibitors have been conjugated with antibodies, which are called ADCs (antibody–drug conjugates), with the aim of specifically targeting the drug to the tumour while avoiding damage to healthy tissues. To date, the antibodies with which inhibitors have been conjugated are antibodies directed against c-kit (receptor tyrosine kinase), which acts as a proto-oncogene in many types of cancer and is involved in processes related to CSCs. Some NAMPT-ADC1–4 conjugates have shown a high efficacy in vivo, so they could be good candidates to enter clinical trials [150,151]. Another targeted therapeutic strategy is called photoactivated chemotherapy (PACT), in which drugs are activated after irradiation with visible light in the tumour. Complexes of ruthenium with NAMPT inhibitors have been developed and have been shown to be effective in hypoxic parts of the tumour since the activation process of the prodrug is independent of oxygen concentration [152]. However, all these new NAMPT inhibitors require further preclinical study before entering clinical trials.

A few years ago, the inhibitor KPT-9274/ATG-019 entered phase 1 clinical trials for patients with advanced solid tumours or Hodgkin’s lymphoma (clinicaltrials.gov, Accessed on 1 April 2022; NCT02702492). This trial evaluated the clinical efficacy of the drug in monotherapy and in combination with niacin (vitamin B3) to palliate the toxic effects and in combination with nivolumab, a monoclonal antibody directed at PD1 (programmed death receptor of T lymphocytes). Research continued in a second phase 1 trial (clinicaltrials.gov; NCT04281420) that is still recruiting patients. In late 2021, KPT-9274 entered a new phase 1 trial to evaluate its efficacy as a monotherapy in patients with relapsed or refractory acute myeloid leukaemia (clinicaltrials.gov, Accessed on 1 April 2022; NCT04914845). Initially, KPT-9274 was discovered as an inhibitor of the PAK4 protein, which is involved in several oncogenic signalling pathways, such as Erk, Akt, Ras/MAPK and Wnt/β-catenin. Later, it was observed that KPT-9274 was also capable of inhibiting NAMPT, which is why it has been proposed as a candidate drug in tumours that express high levels of PAK4 and NAMPT [132,157,158,159,160]. OT-82 is another new NAMPT inhibitor that entered phase 1 clinical trials in 2019 to assess the safety and efficacy in patients with relapsed or refractory lymphoma (clinicaltrials.gov, Accessed on 1 April 2022; NCT03921879). In preclinical trials, OT-82 has shown better results in haematologic malignancies than in solid tumours. OT-82 enhances the efficacy of other drugs, such as cytarabine and dasatinib. Studies in mice and nonhuman primates indicated that OT-82 was well tolerated and showed no cardiac, neurological or retinal toxicity. Resistance to this drug was related to the high expression of CD38 in acute lymphocytic leukaemia cells [114,161].

## Figures and Tables

**Table 1:** NAMPT inhibitors in clinical trials.

Ph	Drug	Type	Condition	ClinicalTrials.gov Identifier	Treatment	Name of the Study
II	**FK866**	Non- competitive	Melanoma	NCT00432107	Alone	Study to Assess APO866 for the Treatment of Advanced Melanoma (2006, completed)
			Cutaneous T-cell Lymphoma	NCT00431912	Alone	Study of APO866 for the Treatment of Cutaneous T-cell Lymphoma (2007, completed)
			B-cell Chronic Lymphocytic Leukemia	NCT00435084	Alone	Study to Assess the Safety and Tolerability of APO866 for the Treatment of Refractory B-CLL (2007, completed)
I	**GMX1778/CHS-828**	Oral competitive	Solid tumors	NCT00003979	Alone	CHS 828 in Treating Patients with Solid Tumors (1999, Withdrawn)
I	**OT-82**	Oral	Relapsed or refractory lymphoma	NCT03921879	Dose escalation and expansion	Safety and Efficacy of OT-82 in Participants with Relapsed or Refractory Lymphoma (2019, recruiting)
I	**KPT-9274/ATG-019**	Non- competitive oral dual inhibitor of PAK4 and NAMPT	Solid tumors, non-Hodgkin’s lymphoma	NCT02702492	Alone or co-administered with Niacin or Nivolumab	PAK4 and NAMPT in Patients with Solid Malignancies or Non-Hodgkin’s Lymphoma (2016, terminated)
			Solid tumors, non-Hodgkin’s lymphoma	NCT04281420	Alone or co-administered with Niacin	Study of Evaluating Dual Inhibitor of PAK4 and NAMPT ATG-019 in Advanced Solid Tumors or Non-Hodgkin’s Lymphoma (2020, recruiting)
			Acute Myeloid Leukemia	NCT04914845	Alone	KPT-9274 in Patients with Relapsed and Refractory Acute Myeloid Leukemia (2021, recruiting)

Ph: clinical phase.

**Table 2:** NAMPT inhibitors in preclinical studies.

Preclinical Drugs	Type	IC50	In Vivo Treatments
**GNE617** **GNE618** **GNE643** **GNE875**	Oral competitive	5 nM	20–30 mg/kg orally in mice [131,133]
**STF-118804**	Competitive	<10 nM	50 mg/kg by subcutaneous injections in mice [135]
**Nampt-IN-1/LSN3154567**	Competitive	3.1 nM	2 mg/kg in mice, 1–2.5 mg/kg or 5 mg/kg (with NA) in dogs [137]
**CB30865** **CB300919**	Competitive	1–10 nM	0.25 mg/kg by intraperitoneal injection in mice [130]
**LB-60-OF61**	Competitive	30 nM	In MYC-overexpressing cell lines [138]
**GPP78/CAY10618**	Competitive	3 nM	10 mg/kg by intraperitoneal injection in mice [139]
**Compound 30**	Competitive	0.13–25.3 nM	15 mg/kg by intravenous injection [140]
**TP201565**	Competitive		In several human cell lines [136]
**MV78**	Competitive	3.1 nM	[141]
**trans-3-(pyridin-3-yl) acrylamide- sulfamides**	Competitive	0.2–5 nM	In several human cell lines [142]
**MPC-9528 MPI0479883**	Competitive	0.06 nM	75 mg/kg in mice [134]
**A-1293201** **A-1307138**	Oral Competitive	11–900 nM	7.5, 15 or 30 mg/kg orally in mice [143,144]
**MS0-MS31**	Competitive	0.9–96 nM	In some human cell lines [145,146]
**STF-31**	Dual inhibitor of GLUT1 and NAMPT	1 µM	In several human cell lines [147]
**Nampt-IN-3**	Dual inhibitor of NAMPT and HDAC	31–55 nM	25 mg/kg by intraperitoneal injection in mice [148]
**Nampt-IN-5**	Dual inhibitor of NAMPT and CYP3A4	0.7–3.9 nM	5–30 mg/kg oral gavage in mice [149]
**NAMPT inhibitor-ADC1–4**	Drug-linker conjugates for ADC (anti-c-Kit)	0.1 pM– 10 nM	3–10 mg/kg by intraperitoneal injection in mice [150,151]
**Water-soluble ruthenium complexes**	Pro-drug photoactivated chemotherapy (PACT)		In skin and lung human tumour cell lines [152]
**Niraparib/Olaparib +NAMPT inhibitors**	Combination with synergistic effect		50 mg/kg orally in mice (PARPi) [153]

## Data Availability

No datasets were generated during the current study.

## Abbreviations

2-HNA: 2-hydroxynicotinic acid; aa: amino acid; ACMS: 2-amino-3-carboxymuconic acid semialdehyde; ACMSD: 2-amino-3-carboxymuconic acid semialdehyde decarboxylase; ADCs: antibody-drug conjugates; ADP-ribose: adenosine diphosphate ribose; AMP: adenosine monophosphate; cADPR: cyclic ADP-ribose; cADPRSs: CCND1: cyclin D1; HNSCC: head and neck squamous cancer; CRISPR-Cas9: clustered regularly interspaced short palindromic repeats—associated with the Cas9 protein; CSCs: cancer stem cells; CXCR4: CXC chemokine receptor type 4; D-2HG: D-2-hydroxyglutarate; DLT: dose limiting toxicity; EGFR: epidermal growth factor receptor; EMT: epithelial-mesenchymal transition; eNAMPT: extracellular NAMPT or visfatin; ENTs: equilibrative nucleoside transporters; G6PDH: glucose-6-phosphate dehydrogenase; GAPDH: glyceraldehyde-3-phosphate dehydrogenase; GSH: glutathione; GSSG: glutathione disulfide; HDAC: histone deacetylase; HIF-1α: hypoxia-inducible factor 1 alpha subunit; HNSCC: head and neck squamous cell carcinoma; IC50: half of the maximum inhibitory concentration; IDH1/2: isocitrate dehydrogenase 1 and 2; IGF-R1: insulin-like growth factor receptor 1; IL-6: interleukin 6; IL-7: interleukin 7; IP: intraperitoneal route; iPSCs: induced pluripotent cells; LDH: lactate dehydrogenase; Mg^2+^: magnesium; NA: nicotinic acid NAAD: nicotinic acid adenine dinucleotide; NAADP: nicotinic acid adenine dinucleotide phosphate; NAD+: nicotinamide adenine dinucleotide; NADH: reduced nicotinamide adenine dinucleotide; NADK: NAD kinase; NADP+: nicotinamide adenine dinucleotide phosphate; NADPH: reduced nicotinamide adenine dinucleotide phosphate; NADSYN: NAD synthetase; NAM: nicotinamide; NAMN: nicotinic acid mononucleotide; NAMPT: nicotinamide phosphoribosyltransferase; NAPRT: nicotinic acid phosphoribosyltransferase; NAR: nicotinic acid riboside; NF-κB: nuclear factor kappa light chain enhancer of activated B cells; NGS: next generation sequencing; NMN: nicotinamide mononucleotide; NMNAT: nicotinamide mononucleotide adenylyltransferase; NNMT: nicotinamide N-methyltransferase; NR: nicotinamide riboside; NRK: nicotinamide ribose kinase; OSKM: Yamanaka factors; OXPHOS: oxidative phosphorylation; PA: picolinic acid; PACT: photoactivated chemotherapy; PARPs: poly-(ADP-ribose); PBEF: pre-B colony-stimulating factor; PD1: programmed death receptor of T lymphocytes; PnC1: nicotinamidase; PPi: pyrophosphate; PPM1D: protein phosphatase 1D; PPP: pentose phosphate pathway; pRb: retinoblastoma protein; PRPP: phosphoribosyl pyrophosphate; QA: quinolinic acid; QPRT: quinolinate phosphoribosyltransferase;ROS: reactive oxygen species; SD: standard deviation; SDF-1: stromal cell-derived factor 1; SCF: stem cell factor; SIRTs: sirtuins; SNP: single nucleotide polymorphism; TCGA: The Cancer Genome Atlas Program; Trp: tryptophan; VHL: von Hippel-Lindau.
